# Converting habits of antibiotic use for respiratory tract infections in German primary care – study protocol of the cluster-randomized controlled CHANGE-3 trial

**DOI:** 10.1186/s13063-019-3209-7

**Published:** 2019-02-06

**Authors:** Anja Wollny, Attila Altiner, Tonia Brand, Katharina Garbe, Martina Kamradt, Petra Kaufmann-Kolle, Mirko Leyh, Regina Poß-Doering, Joachim Szecsenyi, Lorenz Uhlmann, Arwed Voss, Dorothea Weber, Michel Wensing, Christin Löffler

**Affiliations:** 1 Institute of General Practice, Rostock University Medical Center, Rostock, Germany; 2 Department of Medical Biometry, Institute of Medical Biometry and Informatics, University Hospital Heidelberg, Heidelberg, Germany; 3 Department of Communication Design and Media, University of Applied Sciences, Technology, Business and Design, Wismar, Germany; 4 aQua-Institute for Applied Quality Improvement and Research in Health Care GmbH, Göttingen, Germany; 5 Department of General Practice and Health Services Research, University Hospital Heidelberg, Heidelberg, Germany

**Keywords:** Antibacterial agents, Drug resistance, Drug prescriptions, Respiratory tract infections, Primary health care, Randomized controlled trial

## Abstract

**Background:**

The overuse of antibiotics is a major cause for the worldwide rise of antibiotic resistance. Although it is well known that acute respiratory tract infections (ARTI) are mainly caused by viruses and are often self limiting, antibiotics are too frequently prescribed in primary care. CHANGE-3 examines whether a complex intervention focusing on improving communication and provision of prescribing feedback reduces antibiotic use in patients suffering from ARTI.

**Methods/design:**

The CHANGE-3 trial is a cluster-randomized controlled trial nested within a web-based public campaign conducted in two regions in Germany. A total of 114 medical practices will be included. Practices randomized to the intervention will receive a practice-specific antibiotic-prescription feedback and an educational outreach visit. During the visit the whole practice team will receive an introduction to e-learning modules addressing patient-centered communication on antibiotics. Furthermore, the practices will receive tablet PCs with information on antibiotics and the treatment of ARTI to be presented to patients. Practices randomized to the control will provide care as usual. The primary outcome measure is the antibiotic prescribing rate for patients with a history of ARTI. Data collected before the intervention, during the intervention and after the intervention will be compared. The use of narrow- vs. broad-spectrum antibiotics will be analyzed as a secondary outcome. A process evaluation is also part of the trial.

**Discussion:**

This study should contribute to the growing body of research on reducing antibiotic prescription.

**Trial registration:**

ISRCTN, ISRCTN15061174. Registered retrospectively on 13 July 2018.

**Electronic supplementary material:**

The online version of this article (10.1186/s13063-019-3209-7) contains supplementary material, which is available to authorized users.

## Background

The excessive use of antibiotics in human and veterinary medicine is responsible for the worldwide rise of antibiotic resistance [1, 2]. In Germany, approximately 40–45% of all antibiotics prescribed in human medicine stem from primary care, with acute respiratory tract infections (ARTI) [3] being the most common cause. Although among both experts and non-professionals it is well known that ARTI are primarily caused by viruses and are mostly self-limiting, in too many cases antibiotics are prescribed [4–6]. The main reasons for the inadequate prescription of antibiotics in primary care include a misleading safety culture and general practitioners’ (GPs) misconception that patients would expect an antibiotic prescription [7, 8]. This leads to over-prescribing of broad-spectrum antibiotics such as quinolones and cephalosporins [3]. This eventually leads to increasing rates of antibiotic resistance, avoidable side effects and drug interactions as well as unnecessary direct and indirect costs, e.g., for hospital admissions [9–13].

Since the 1990s, efforts to reduce unnecessary antibiotic prescriptions in primary care have been made [3, 14]. Trials have proven that some interventions are able to lower medication prescription rates in a relevant manner [15, 16]. However, the effect of interventions is varying and is specific to situations and conditions [17]. A recent systematic review on interventions aiming at reducing antibiotic prescription for ARTI patients showed, for instance, positive effects of single-element and multifaceted interventions using GP communication skills’ training and point-of-care testing. Other elements, such as patient-centered information alone, had no effect [16]. Wensing et al. showed efficacy of quality circles and written feedback on professional performance and prescribing [18]. So far, most interventions focus on GPs [15, 19]. Research has shown that sensitizing the practice staff for a better interaction with patients carries the potential to relieve the GP’s work and to contribute to improved decision-making in general [20, 21]. Practice staff can play an important role in transferring knowledge to patients [21]. Thus, the idea of addressing the whole practice team in interventions to reduce unnecessary antibiotic prescriptions is gaining momentum.

## Aim

The cluster-randomized controlled CHANGE-3 trial (cRCT) aims at testing the effect of a practice intervention including practice-specific antibiotic-prescription feedback, an education outreach visit, patient-centered communication, and patient information on the rate of antibiotic prescription for ARTI in German primary care.

Patient-centered communication will be conveyed via e-learning modules. Versions for physicians and practice staff members will differ and will be target-group-specific.

For the delivery of patient information, practices will receive tablet PCs with multimedia patient information on antibiotics and the treatment of ARTI.

The cRCT is nested within a larger-scale, web-based, public campaign conducted in two German states and has the title “*Weniger Antibiotika: mehr Ideen*” (English; less antibiotics: more ideas) [22]. Whereas the campaign tests the effectiveness of informing the patient and, to some extent, health care provider information at large, the cRCT investigates whether this intensified approach produces further effects. GP practices of the control group will provide care as usual, but will have access to the web-based campaign just like the general public.

## Methods/design

### Trial design

**Fig. 1:**
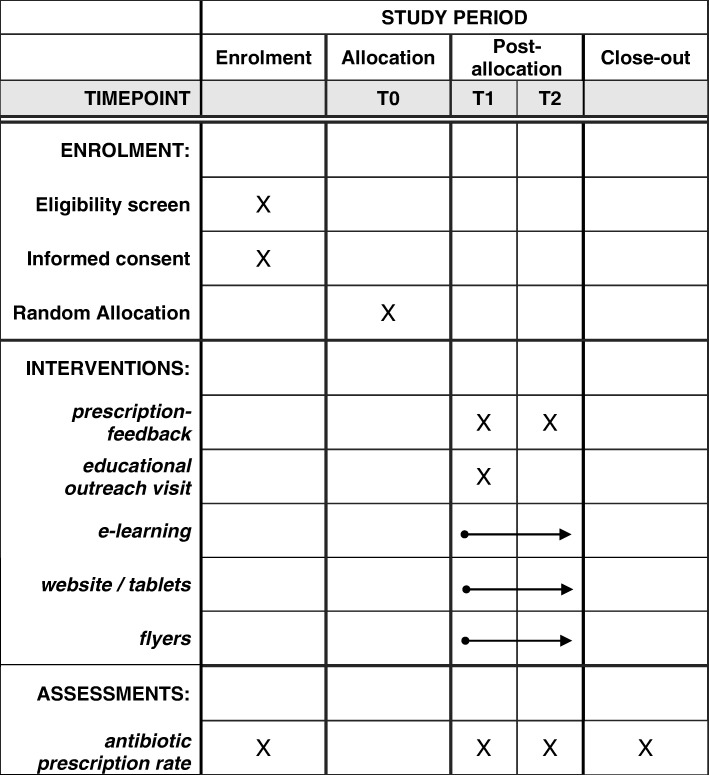
Overview of enrollment, intervention, and assessments of the CHANGE-3 trial

CHANGE-3 is a two-arm cRCT with GP practices and their patients forming a cluster. It is nested within a web-based public campaign conducted in two German regions. The antibiotic prescription rates of participating GPs will be measured over two successive winter seasons (Fig. 1). Additionally, the quality of prescriptions in terms of active ingredient chosen (broad- vs. narrow-spectrum antibiotics), usage of quinolones, seasonal fluctuations, and the knowledge of patients about antibiotics and treatment of ARTI will be monitored. GP practices will be allocated randomly either to the intervention group (IG) or the control group (CG). In the latter, care will be provided as usual. Claims data from the largest German statutory health insurance provider, AOK, will be used for all analyses. The SPIRIT checklist for this protocol can be found in Additional file 1.

### Recruitment

GP practices will be recruited in the two German states, Mecklenburg-Western Pomerania (MV) and Baden-Wurttemberg (BW). A total of 114 practices will be included in the trial (38 GPs in MV and 76 GPs in BW). GPs will be approached via invitation letters. Interested physicians will receive trial information via telephone or during a practice visit. Participating GPs will provide informed consent in writing. They will also agree to release their patients’ anonymized prescription data as stored by the German statutory health insurance provider AOK (§§295, 300 SGB V). These data include all AOK-insured patients diagnosed with ARTI who receive medical treatment during the trial. Using these data, overall and practice-specific antibiotic prescription rates will be determined.

In addition, two written patient surveys will be conducted over two successive winter seasons. In each survey participating GP practices will recruit up to 50 ARTI patients aged 17 years or over to fill in a questionnaire. The questionnaire will measure patients’ knowledge of antibiotics as well as expectations and experiences related to antibiotic prescribing. Patients’ participation in the survey will be anonymous and not related to any affiliation with a specific health insurance provider. Exclusion criteria are: not being able to read or fill in the questionnaire.

### Intervention

The intervention is multifaceted and includes different elements (Additional file 2). The IG will receive feedback on practice-specific antibiotic prescription on two occasions (Additional file 2, component 1) and an educational outreach visit (Additional file 2, component 2), which is a central focus of the intervention. During the visit, a specially trained expert will discuss the practice-specific antibiotic-prescription feedback provided in detail. Additionally, the practice will have access to e-learning modules focusing on communication strategies, e.g., on how to handle patients expecting the prescription of antibiotics (Additional file 2, component 3 and 4). Patient information on the common cold, middle ear infections, and sinus infections is tailored to different groups of patients, e.g., the elderly or parents of children with ARTI, and is provided in the form of flyers and information on tablet computers (Additional file 2, components 5 and 6). Communication designers will be involved in the development of the materials.

### Control

Patients of the CG will receive care as usual. Since the cRCT will take place during the web-based public campaign, GP practices of the CG will have access to the campaign’s materials as the general public does.

### Data collection, completeness and quality

Recruitment of GPs started in August 2017 and will continue until September 2018. The anonymized prescribing data from the GPs’ patients will be retrieved at the beginning of the trial (baseline, T0) and over two successive winter seasons: during the intensified intervention phase in the first winter season (T1) and the second winter season (T2). That means that baseline data will be retrieved retrospectively after provision of written informed consent by GPs. The intervention will start after baseline collection is completed and continue throughout the trial, due to the public campaign running in parallel. To identify participating GPs in the secondary health data from AOK, two GP identifiers will be collected (“*Lebenslange Arztnummer*”; LANR and “*Betriebsstättennummer*”; BSNR). In addition, the following information will be gathered at the start of or during the trial: age and gender of GP, time since practice setup, specialist title of GP, and practice characteristics, like number of team members and field of expertise.

Also, up to 50 patient questionnaires on patient knowledge of antibiotics and treatment of ARTI will be delivered by the GPs. Participation of ARTI patients is voluntary and anonymous. This will be done throughout the entire trial. The questionnaires will collect information on patients’ age and gender, employment situation, first language, educational background, knowledge on antibiotics, and reason for consultation. To avoid duplicates, participants who fill out the questionnaire will be listed anonymously (date of questionnaire dissemination, year of birth, and gender).

### Randomization

Participating GP practices will be randomly assigned in a 1:1 ratio into the two study groups after recruitment. Randomization will be stratified by rate of antibiotic prescription for ARTI at baseline per practice. Randomization will be performed by the Institute of Medical Biometry and Informatics at the University Hospital Heidelberg.

### Outcome measures

The primary outcome measure is the GPs’ antibiotic prescription rate for ARTI at the end of the trial in comparison with the baseline data. These outcome data come from routinely collected data that are provided by the German statutory health insurance provider AOK. Tables 1 and 2 summarize relevant Anatomical Therapeutic Chemical (ATC) and International Classification of Diseases (ICD) codes, respectively, for calculating antibiotic prescription rates.

**Table 1:** Relevant Anatomical Therapeutic Chemical (ATC) codes

Drug	ATC code	Drug group	Group name
Definition of classified ATC codes	J01FA (without J01FA01)	Macrolides (without erythromycin) – *broad-spectrum antibiotics*	Macrolides
	J01DB	First-generation cephalosporins	Cephalosporins
	J01 DC	Second-generation cephalosporins	Cephalosporins
	J01DD	Third-generation cephalosporins	Cephalosporins
	J01DE	Fourth-generation cephalosporins	Cephalosporins
	J01MA	Fluoroquinolones	Gyrase inhibitors
	J01CR	Penicillins including beta-lactam inhibitors *– broad-spectrum antibiotics*	Penicillins + beta-lactam-inhibitors
	J01CA	Penicillins with extended spectrum	Penicillins with extended spectrum
	J01 CE	Beta-lactamase-sensitive penicillins	Beta-lactamase-sensitive penicillins
	J01EE01	Sulfamethoxazole and trimethoprim	Sulfamethoxazole and trimethoprim
	J01XE01	Nitrofurantoin	Trimethoprim, nitrofurantoin, fosfomycin
	J01EA01	Trimethoprim	Trimethoprim, nitrofurantoin, fosfomycin
	J01XX01	Fosfomycin	Trimethoprim, nitrofurantoin, fosfomycin
	J01FA01	Erythromycin	Erythromycin
	J01FA16	Solithromycin	Erythromycin
	J01A	Tetracyclines	Tetracyclines
	J01other	J01-other	Other antibiotics

**Table 2:** Relevant International Classification of Diseases, version 10 (ICD-10) codes

Diagnoses	ICD-10	Indication	ICD group
Definition of classified ICD-10 codes	J20; J21.0; J21.1; J21.8; J21.9; J22; J40	Acute bronchitis	Lower respiratory infection
	J00; J02.0; J02.8; J02.9; J04; J06; J10.1; J11.1	Acute infection of the upper respiratory tract	Upper respiratory infection
	J01	Acute sinusitis	Upper respiratory infection
	J03.0; J03.8; J03.9	Tonsillitis	Upper respiratory infection
	H65.0; H65.1; H65.9; H66.0; H66.4; H66.9	Otitis media	Upper respiratory infection

The secondary outcome is the choice of active ingredient (broad- vs. narrow-spectrum) in the antibiotic prescribed.

Additionally, data for the outcomes ‘patients’ level of knowledge on antibiotics’ and ‘patients’ expectations and experiences related to antibiotic prescribing’ are collected via questionnaires in two written patient surveys. The surveys’ results are meant as a feedback to the practices about their patients’ perspective. The results of the second patient survey will show, in comparison with the first ones, whether the project interventions resulted in changes concerning patients’ expectations and patients’ knowledge. Descriptive statistics and specific tests for group comparison will be used to analyze survey data (IBM SPSS statistics 20).

### Methods to reduce bias and enhance data quality

With reference to the Cochrane Collaboration’s tool for assessing risk of bias [23], a number of procedures are carried out to reduce potential sources of bias. Firstly, to reduce selection bias, participating GP practices in both states are recruited with identical procedures. A non-responder analysis will also be performed. Secondly, randomization of participating practices will be performed electronically by statisticians not involved in physician recruitment. Thirdly, specified inclusion and exclusion criteria (ICD and ATC codes) will be used to calculate antibiotic prescription rates for ARTI among participating practices in order to reduce the risk for information bias and selective outcome reporting. Fourthly, to reduce variation in intervention delivery, outreach visits will be standardized. Due to financial and time constraints within this trial it will not be possible to blind participants and trial staff. Prior to its start, the study was registered in the public trial archive ISRCTN.

### Sample size calculation

**Fig. 2:**
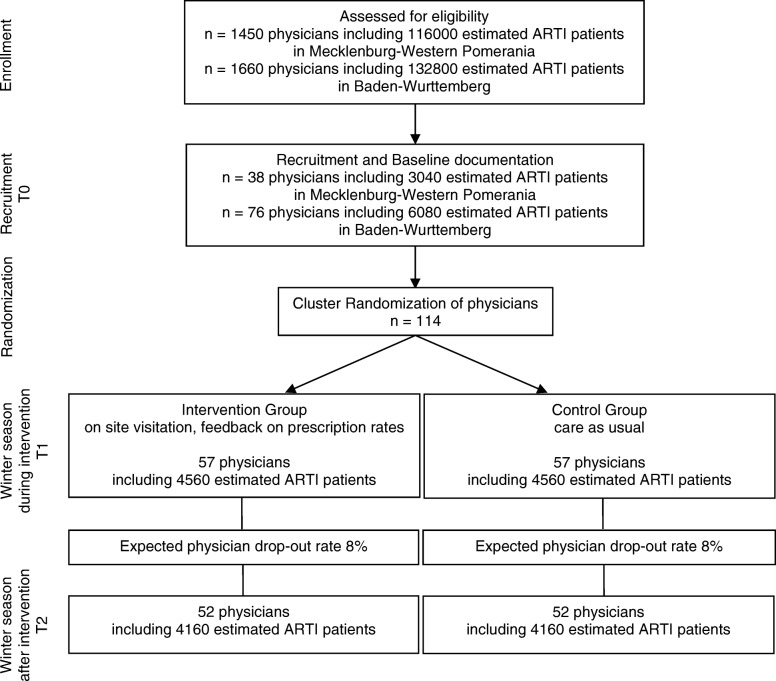
Flow chart of the CHANGE-3 trial

We expect to find a relative reduction of the antibiotic prescription rate of 30% (which means an absolute reduction from 40 to 28%) between the groups. The sample size considerations are based on the chi-squared test using a significance level of α = 5% (two-sided) and a power of (1 − β) = 80%. This results in a sample size of *n* = 244 patients per group. To take the clustered structure into account, we assumed an intra-cluster correlation coefficient (ICC) for patients in practices of 0.2 [24]. This value is based on previous studies. The cluster size is assumed to be *m* = 80 patients. Thus, the design effect (*DE*) equals *DE* = 1 + (*m* − 1) × *ICC* = 1 + 79 × 0.2 = 16.8. These considerations result in a sample size of *N* = *n* × *DE* = 244 × 16.8 = 4100 patients per group. Thus, the total sample size is 104 practices of size *m* = 80 resulting in 8320 patients. A drop-out rate of about 8% is expected and, therefore, 10 additional practices will be recruited (see Fig. 2 for detailed information on the scheduled flow of patients). The analysis will be done by using a logistic mixed-effects model, which we expect to explain more of the variance in the prescription of antibiotics. Therefore, the model should be more powerful than the chi-squared test. The calculations are done using SAS 9.4 (Proc Power).

### Statistical analyses

In a first step, descriptive statistics will be provided for the primary and secondary outcomes as well as for patient, practice, and GP characteristics, including mean, standard deviation, median, interquartile range (IQR), minimum and maximum for continuous variables, and frequency in percentages for categorical variables. The primary objective of this study is to examine the difference in antibiotic prescription rate after intervention (T1) between the two study arms.

The confirmatory analysis of the primary endpoint will be conducted based on the intention-to-treat (ITT) population, where all patients will be included in the analysis and assigned to the group they were randomized to.

A logistic mixed-effects model will be applied to assess the respective hypothesis of equal rates in both groups regarding the primary endpoint using a significance level of α = 5%. A random intercept will be included for practices. Furthermore, age and gender of patients will be included as covariates. Missing values regarding the primary endpoint will be imputed using multiple imputation. As a sensitivity analysis, the primary endpoint will be evaluated on the per-protocol population, where patients with major protocol violations will be excluded. As a further sensitivity analysis, the baseline prescription rate on practice level will be included as an additional covariate in the primary logistic mixed-effects model.

Mixed models, as described for the primary endpoint, will be used to analyze the secondary outcomes. All calculated *p* values regarding secondary endpoints will be of a descriptive nature. A detailed statistical analysis plan will be written prior to the final analysis. SAS (SAS Institute Inc., Cary, NC, USA) version 9.4 or higher will be used to carry out the analyses. A non-responder analysis is planned regarding size of practice, number of antibiotic prescriptions in case of ARTI, and application of broad-spectrum antibiotics.

### Process evaluation

To understand the mechanisms of the educational components, a process evaluation is accompanying the intervention in the CHANGE-3 study. Using a mix of qualitative and quantitative research methods, the process evaluation aims at assessing reach and fidelity of the implementation program, its effects on daily practice of health care delivery to ARTI patients and the impact of diverse context factors on health services. The survey as well as the interview guide used within the process evaluation are based on the Theoretical Domains Framework (TDF) [25, 26] to gain insight into working mechanisms. The survey focuses on aspects regarding the regional intervention with multimedia content (web-based public campaign), the practice team intervention with outreach visit, e-learning and feedback report and on personal perceptions regarding the provision of health services to ARTI patients. Interviews will add in-depth understanding of educational intervention components, context factors impacting on the uptake of the intervention and the significance and role of the implemented set of measures to practice teams and patients.

In the IG, data collection will occur through telephone and face-to-face interviews with GPs, medical assistants (GP practice nurses), patients, and outreach visitors as well as surveys for health care providers. In the CG, patient interviews and provider surveys will be used to collect data. Survey data will be collected twice. All physicians and medical assistants in the IG and CG will be invited to participate in the process evaluation. A purposive sampling strategy will be followed to recruit interview participants among GPs and medical assistants (*n* = 20), patients (*n* = 20), and outreach visitors (*n* = 5).

Interviews will be recorded and transcribed. The pre-defined categories of a framework, such as Flottorp et al. [27] or Atkins et al. [26], will be used to identify determinants of practice which influence health care delivery to ARTI patients in general practices, with regard to the educational components of the intervention and with regard to converting habits of antibiotic use. Descriptive statistics as well as specific tests for group comparison will be used to analyze survey data. Further analyses which investigate the relationship between survey-based and claims-data-based outcomes will be applied if the sample size is sufficiently large. All collected data will be pseudonymized for analysis.

The results of the process evaluation will strengthen the reporting in CHANGE-3 and provide information for future implementation programs.

## Discussion

As a cRCT, CHANGE-3 aims at improving antibiotic prescription among ARTI patients cared for in general practice. The cRCT is nested in a large public campaign that is geared towards sensitizing the population of two German states for the reasonable use of antibiotics.

CHANGE-3 is based on two previous studies (CHANGE and CHANGE-2) investigating the efficiency of improved physician-patient communication, patient empowerment and point-of-care testing on antibiotic prescription rates [19, 24]. The study benefits from lessons learnt from these previous trials and, beyond that, includes recent findings on the importance of patient information, on a practice-based approach and on the effect of practice-specific prescription feedback [28, 29]. CHANGE-3 is designed to test the hypothesis that a relative reduction of 30% in antibiotic prescription can be achieved by comparatively simple means.

### Trial status

Recruitment of GPs will be finished in September 2018. Recruitment of patients is ongoing throughout the trial.

### Trial registration

The trial is registered at ISRCTN under the reference ISRCTN15061174. Registered retrospectively on 13 July 2018; http://www.isrctn.com/ISRCTN15061174.

## Additional files

Additional file 1: SPIRIT checklist: recommended items to address in a clinical trial protocol. (DOCX 64 kb)

Additional file 2: Intervention description according to Template for Intervention Description and Replication (TIDieR) [30]. (DOCX 96 kb)

## Abbreviations

ARTI: Acute respiratory tract infections
BSNR: “*Betriebsstättennummer*” is a unique number which identifies every practice in Germany
BW: Baden-Wurttemberg
CG: Control group
DE: Design effect
GP: General practitioner
ICC: Intra-cluster correlation coefficient
IG: Intervention group
IQR: Interquartile range
ITT: Intention-to-treat
LANR: “*Lebenslange Arztnummer*” is a unique identification number for each physician in Germany
MV: Mecklenburg-Western Pomerania

## References

[1] Bundesministerium für Gesundheit. DART 2020. 2015. https://www.bmel.de/SharedDocs/Downloads/Broschueren/DART2020.pdf?__blob=publicationFile. Accessed 18 July 2018.

[2] Roca I, Akova M, Baquero F, Carlet J, Cavaleri M, Coenen S, et al. The global threat of antimicrobial resistance: science for intervention. New Microbes New Infect. 2015;6:22–9. 10.1016/j.nmni.2015.02.007 PMC4446399 26029375

[3] Bundesamt für Verbraucherschutz und Lebensmittelsicherheit (2017). Germap 2015.

[4] KBV. Rationale Antibiotikatherapie bei Infektionen der oberen Atemwege. In: Wirkstoff AKTUELL. 2012. https://www.akdae.de/Arzneimitteltherapie/WA/Archiv/Antibiotika-URTI.pdf. Accessed 18 July 2018.

[5] KBV. Rationale Antibiotikatherapie bei Infektionen der unteren Atemwege. In: Wirkstoff AKTUELL. 2017. https://www.akdae.de/Arzneimitteltherapie/WA/Archiv/Antibiotika-LRTI.pdf. Accessed 18 July 2018.

[6] Altiner A, Bell J, Duerden M, Essack S, Kozlov R, Noonan L, et al. More action, less resistance: report of the 2014 Summit of the Global Respiratory Infection Partnership. Int J Pharm Pract. 2015;23(5):370–7. 10.1111/ijpp.12177 25711969

[7] Kumar S, Little P, Britten N. Why do general practitioners prescribe antibiotics for sore throat? Grounded theory interview study. BMJ. 2003;326(7381):138. 10.1136/bmj.326.7381.138 PMC140007 12531847

[8] Petursson P. GPs’ reasons for “non-pharmacological” prescribing of antibiotics. A phenomenological study. Scand J Prim Health Care. 2005;23(2):120–5. 10.1080/02813430510018491 16036552

[9] Höffken G, Lorenz J, Kern W, Welte T, Bauer T, Dalhoff K, et al. Epidemiology, diagnosis, antimicrobial therapy and management of community-acquired pneumonia and lower respiratory tract infections in adults. Guidelines of the Paul-Ehrlich-Society for Chemotherapy, the German Respiratory Society, the German Society for Infectiology and the Competence Network CAPNETZ Germany. Pneumologie. 2009;63(10):e1–68. 10.1055/s-0029-1215037 19821215

[10] Schröder H. Hände weg von der eisernen Reserve. Gesundheit und Gesellschaft. 2012;7-8/11:21–7.

[11] Del Mar CB, Glasziou PP, Spinks AB. Antibiotics for sore throat. Cochrane Database Syst Rev. 2006;(4):CD000023. 10.1002/14651858.CD000023.pub3 17054126

[12] German College of General Practitioners and Family Physicians (DEGAM). Halsschmerzen DEGAM-Leitlinie Nr. 14. 2009. https://www.degam.de/files/Inhalte/Leitlinien-Inhalte/Dokumente/DEGAM-S3-Leitlinien/Leitlinien-Entwuerfe/053-010_Halsschmerzen/LL-14_Langfassung_ZD.pdf. Accessed 23 Mar 2018.

[13] National Institute for Health and Clinical Excellence. Respiratory tract infections - antibiotic prescribing. NICE clinical guideline 69. 2008. https://www.nice.org.uk/guidance/cg69/evidence/full-guideline-196853293. Accessed 23 Mar 2018. 21698847

[14] Bundesinstitut für Arzneimittel und Medizinprodukte, Paul-Ehrlich-Institut. Bulletin zur Arzneimittelsicherheit - Informationen aus BfArM und PEI. 2015. https://www.bfarm.de/SharedDocs/Downloads/DE/Arzneimittel/Pharmakovigilanz/Bulletin/2015/3-2015.pdf?__blob=publicationFile&v=6. Accessed 23 Mar 2018.

[15] Coxeter P, Del Mar CB, McGregor L, Beller EM, Hoffmann TC. Interventions to facilitate shared decision making to address antibiotic use for acute respiratory infections in primary care. Cochrane Database Syst Rev. 2015;(11):CD010907. 10.1002/14651858.CD010907.pub2 PMC6464273 26560888

[16] Köchling A, Löffler C, Reinsch S, Hornung A, Böhmer F, Altiner A, Chenot J-F. Reduction of antibiotic prescriptions for acute respiratory tract infections in primary care: a systematic review. Implement Sci. 2018;13(1):47. 10.1186/s13012-018-0732-y PMC5859410 29554972

[17] Arnold SR, Straus SE. Interventions to improve antibiotic prescribing practices in ambulatory care. Cochrane Database Syst Rev. 2005;(4):CD003539. 10.1002/14651858.CD003539.pub2 PMC7003679 16235325

[18] Wensing M, Broge B, Riens B, Kaufmann-Kolle P, Akkermans R, Grol R, et al. Quality circles to improve prescribing of primary care physicians. Three comparative studies. Pharmacoepidemiol Drug Saf. 2009;18(9):763–9. 10.1002/pds.1778 19507170

[19] Altiner A, Berner R, Diener A, Feldmeier G, Köchling A, Löffler C, et al. Converting habits of antibiotic prescribing for respiratory tract infections in German primary care—the cluster-randomized controlled CHANGE-2 trial. BMC Fam Pract. 2012;13:124. 10.1186/1471-2296-13-124 PMC3548682 23256712

[20] Freund T, Peters-Klimm F, Boyd CM, Mahler C, Gensichen J, Erler A, et al. Medical assistant-based care management for high-risk patients in small primary care practices: a cluster randomized clinical trial. Ann Intern Med. 2016;164(5):323–30. 10.7326/M14-2403 26833209

[21] Chapman SA, Blash LK. New Roles for Medical Assistants in Innovative Primary Care Practices. Health Serv Res. 2017;52:383–406. 10.1111/1475-6773.12602 PMC5269548 27859097

[22] Weniger Antibiotika: mehr Ideen; 2018. https://www.weniger-antibiotika.de. Accessed 30 October 2018.

[23] Higgins JPT, Green S (editors). Cochrane handbook for systematic reviews of interventions. John Wiley and Sons Ltd, The Atrium, Southern Gate, Chichester, West Sussex PO19 8SQ, England; 2008.

[24] Altiner A, Brockmann S, Sielk M, Wilm S, Wegscheider K, Abholz HH. Reducing antibiotic prescriptions for acute cough by motivating GPs to change their attitudes to communication and empowering patients: a cluster-randomized intervention study. J Antimicrob Chemother. 2007;60(3):638–44. 10.1093/jac/dkm254 17626023

[25] Francis JJ, O’Connor D, Curran J. Theories of behaviour change synthesised into a set of theoretical groupings: introducing a thematic series on the theoretical domains framework. Implement Sci. 2012;7:35. 10.1186/1748-5908-7-35 PMC3444902 22531601

[26] Atkins L, Francis J, Islam R, O’Connor D, Patey A, Ivers N, et al. A guide to using the Theoretical Domains Framework of behaviour change to investigate implementation problems. Implement Sci. 2017;12(1):77. 10.1186/s13012-017-0605-9 PMC5480145 28637486

[27] Flottorp SA, Oxman AD, Krause J, Musila NR, Wensing M, Godycki-Cwirko M, et al. A checklist for identifying determinants of practice: a systematic review and synthesis of frameworks and taxonomies of factors that prevent or enable improvements in healthcare professional practice. Implement Sci. 2013;8:35. 10.1186/1748-5908-8-35 PMC3617095 23522377

[28] Hallsworth M, Chadborn T, Sallis A, Sanders M, Berry D, Greaves F, et al. Provision of social norm feedback to high prescribers of antibiotics in general practice: a pragmatic national randomised controlled trial. Lancet. 2016;387:1743–52. 10.1016/S0140-6736(16)00215-4 PMC4842844 26898856

[29] Meeker D, Linder JA, Fox CR, Friedberg MW, Persell SD, Goldstein NJ, et al. Effect of behavioral interventions on inappropriate antibiotic prescribing among primary care practices: a randomized clinical trial. JAMA. 2016;315(6):562–70. 10.1001/jama.2016.0275 PMC6689234 26864410

[30] Hoffmann TC, Glasziou PP, Boutron I, Milne R, Perera R, Moher D, et al. Better reporting of interventions: template for intervention description and replication (TIDieR) checklist and guide. BMJ. 2014;348:g1687. 10.1136/bmj.g1687 24609605

